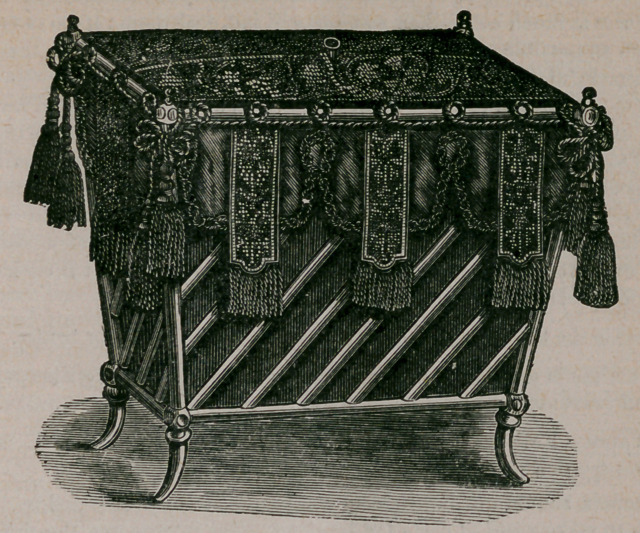# Household

**Published:** 1888-04

**Authors:** 


### HOUSEHOLD.

NEGLIGEE BASKET.—A useful article for a bed-room as a receptacle for ladies' and children's night-clothes. The frame can be made of rustic work if desirable, lined with any colored material; it should have two half covers or lids at the top, which may be embroidered to suit the taste or fancy. The sides may be festooned with silk, ornamented with fringe or other material, with tassels at the corners. It is given as a suggestion to those who enjoy the making of such fancy conveniences.

HOW TO COOK RICE.—Rice is becoming a much more popular article of food than heretofore. It is frequently substituted for potatoes at the chief meal of the day, being more nutritious and much more readily digested. At its present cost, it is relatively cheaper than potatoes, oatmeal, or grain grits of any kind. In preparing it only just enough cold water should be poured on to prevent the rice from burning at the bottom of the pot, which should have a close-fitting cover, and with a moderate fire the rice is steamed rather than boiled until it is nearly done ; then the cover is taken off, the surplus steam and moisture allowed to escape, and the rice turns out a mass of snow-white kernels, each separate from the other, and as much superior to the usual soggy mass as a fine mealy potato is superior to the water-soaked article.

CHEAP SOUP.—Only practical cooks know how little meat is needed to make good soups. The bones of a turkey from which all the meat has been removed make good soup ; also the bones from a roast of beef or mutton do as well. It is better to crack the bones and boil them the day before using the soup, so as to take off every particle of fat. Put turnips, carrots, onions, any kind of vegetables you like in fact, and let all boil together three or four hours ; when ready for the table, have a slice of bread ready toasted, cut into small, square bits, put into a dish and strain the soup over it through a colander.—*Housekeeper.*

STUFFED STEAK.—Take a good slice of steak, about one pound, cut in one piece and pretty thick : then prepare a breakfastcupful of bread or hard scraps of bread and pour over them as much hot milk or water as will just soak them ; if too moist squeeze out as much as possible. Beat it up with a fork to break any lumps, then add one large tablespoonful of suet chopped finely, one tablespoonful of parsley chopped up, one small onion chopped, half a teaspoonful of salt, a little pepper, and mix all well together ; use a little flour to mix into a large ball. Roll it up into a nice round shape with string. Dust all over the outside with flour. Put into a small stewpan one tablespoonful of dripping, and let it get quite melted ; put in the steak and fry it all around carefully till the outside is quite brown, then put in a very little water, perhaps half a teacupful, and cover down the lid ; let it stew very slowly, turning it over often ; add from time to time a little water. Let it cook one hour and take off the string and serve with the gravy over it.

Hot water is the best thing that can be used to heal a sprain or bruise.

A little ammonia and borax in the water when washing blankets keeps the flannel soft and prevents shrinking.

FRIED TRIPE.—Roll the boiled tripe, cut in squares, in egg, then in cracker crumbs, and fry to a nice brown. Serve with catsup.

Housekeepers should not fail to keep a bushel or two of charcoal in the house with which to make a bed of coals for broiling. Try it, and see the difference it will make in your steak or chicken or ham.

A simple remedy for neuralgia is to apply grated horse-radish, prepared the same as for table use, to the temple when the face or head is affected, or to the wrist when the pain is in the arm or shoulder.

A quart of buttermilk and a teaspoonful of saleratus, stirred up with buckwheat flour makes the best pancakes ? When done, steaming them in a covered dish improves them very much.

A NICE WAY TO COOK CHICKEN.—Cut up the chickens, put into pan, cover with water and let stew as usual. When done make a thickening of cream and flour · add butter, pepper and salt. Have ready a nice short cake, baked and cut in squares. Lay the squares on a dish and pour the chicken and gravy over them while hot.

LEMONS may often be used as a good household medicine. They are undoubtedly very excellent for billiousness. Lemons, however, should not be taken in their pure state, as their acidity will injure the teeth and the lining of the stomach. The proper way is to take the juice of one lemon in a cup of water, without sugar. The best time to take such a dose is before breakfast or just before retiring. Lemonade is an excellent drink in summer, and can be used with benefit by everyone.

DRIED APPLE CAKE.—Two cups of sweet dried apples, soak over night and chop ; two cups of molasses, and let it simmer over two hours ; when cold add one cup of sugar, two eggs, one-half cup of sour cream, sour milk and butter, two teaspoonfuls of soda, four cups of flour, four teaspoonfuls of cinnamon, and one teaspoonful of cloves and one nutmeg.

## Figures and Tables

**Figure f1:**